# Dietary Calcium Intake and Fat Mass in Spanish Young Adults: The Role of Muscle Strength

**DOI:** 10.3390/nu13124498

**Published:** 2021-12-16

**Authors:** Ana Torres-Costoso, Vicente Martínez-Vizcaíno, Rubén Fernández-Rodríguez, Irene Sequí-Dominguez, Sara Reina-Gutiérrez, Sergio Núñez de Arenas-Arroyo, Miriam Garrido-Miguel

**Affiliations:** 1 Facultad de Fisioterapia y Enfermería, Universidad de Castilla-La Mancha, 45071 Toledo, Spain; AnaIsabel.Torres@uclm.es; 2 Centro de Estudios Socio-Sanitarios, Universidad de Castilla-La Mancha, 16071 Cuenca, Spain; Ruben.Fernandez@uclm.es (R.F.-R.); irene.sequidominguez@uclm.es (I.S.-D.); Sara.Reina@uclm.es (S.R.-G.); Sergio.NunezdeArenas@uclm.es (S.N.d.A.-A.); Miriam.Garrido@uclm.es (M.G.-M.); 3 Facultad de Ciencias de la Salud, Universidad Autónoma de Chile, Talca 3467987, Chile; 4 Facultad de Enfermería de Albacete, Universidad de Castilla-La Mancha, 02006 Ciudad Real, Spain

**Keywords:** adiposity, calcium, muscular fitness, college student, mediation, Spain, fat mass, adults

## Abstract

Obesity is declared as a chronic multifaceted health problem, and young adults may be particularly vulnerable to weight gain. This study aims to identify the role of dietary calcium intake and the muscle strength index in handling excess of fat mass in young adults and to examine if the relationship between dietary calcium intake and fat mass percentage is mediated by muscle strength. A cross-sectional study including 355 Spanish college students (aged 21.05 ± 3.11) was performed during the 2017–2018 academic year. Pearson correlation coefficients were estimated to determine the relationship between dietary calcium intake, fat mass percentage, body mass index, muscle strength components, and total energy intake. ANCOVA models were used to analyze the differences in the muscle strength index by total dietary calcium intake categories, as well as the differences in % fat mass by total dietary calcium intake and muscle strength index categories, controlling for different sets of confounders. A mediator analysis was conducted to test if the relationship between dietary calcium intake and fat mass percentage was explained by muscle strength. Data on the fat mass percentage, dietary calcium intake, and muscle strength index as the sum of the standardized z-score of the standing long jump and z-score of handgrip/weight were collected. The muscle strength index was significantly better in young adults with higher dietary calcium intake. Moreover, the fat mass percentage was significantly lower in those with a higher dietary calcium intake and a better muscle strength index. Finally, the relationship between dietary calcium intake and fat mass percentage was fully mediated by muscle strength (z = −1.90; *p* < 0.05), explaining 33.33% of this relationship. This study suggests that both a major dietary calcium intake and muscle strength are associated with fat mass percentage. Moreover, muscle strength mediates the link between dietary calcium intake and fat mass percentage. Therefore, both high dietary calcium intake and exercise activities aimed at improving muscle strength levels may help to prevent the cardiometabolic risk associated with an excess of fat mass in young people.

## 1. Introduction

Obesity has been declared a chronic multifaceted health problem that affects 22% of the Spanish population [1]. Childhood and adolescent obesity are related not only to adulthood obesity but also to other obesity-related chronic disorders and cardiovascular mortality and all-cause mortality [2,3]; moreover, young adults may be particularly vulnerable to weight gain [4]. The risk of excess fat mass in this population has been related in part to lifestyle factors such as leaving home, cohabiting, or takeaway food consumption [4,5]. In this critical period, lifestyle interventions related to diet and physical activity seem to have a key role as modifiable factors to control the imbalance of fat mass [6].

Muscle strength is currently recognized as an important marker of health associated with cardiometabolic risk factors [7,8,9]. However, a challenging relationship has been described between adiposity and muscle strength. It is well known that the loading effect of fat mass could be a training stimulus to improve muscle strength in youth [10]. Nevertheless, excessive fat mass may also be linked to a lower muscle quality by altering muscular fibers and disturbing calcium cycling, which could adversely influence muscle strength [11,12]. In addition, low levels of muscular fitness are associated with fat accumulation, and therefore, whether muscle strength is a cause or a consequence of excess adiposity remains to be clarified.

From a nutritional point of view, although the vitamin D/calcium supplementation has not shown a significant effect on muscle strength in young adults [13], it is recognized that calcium plays an integral role in muscle contraction, regulating the interplay of filaments in muscle fibers, as calcium is necessary for cross-bridge formation, and consequently for muscle contraction [14,15]. Moreover, although the influence of macronutrients on adiposity management is commonly accepted, the specific effect of micronutrients has been less explored. Among the different micronutrients, calcium may have a substantial role in fat mass regulation mainly through effects such as increasing thermogenesis, reducing lipogenesis, or inhibiting fat absorption [16,17]. In this sense, previous reviews, cross-sectional and longitudinal studies have proposed that dietary calcium may be inversely associated with adiposity in adults [18], adolescents [19], and children [20] but, there is little evidence in the young population. To date, studies linking dietary calcium, fat mass, and muscle strength are restricted, and none have tested if muscle strength plays a role as confounding or as an intermediate variable in the association between dietary calcium and fat mass.

Accordingly, the purpose of this study was to identify the role of dietary calcium intake and muscle strength in young adults in handling the excess of fat mass in this population and to investigate whether the relationship between dietary calcium intake and fat mass percentage is mediated by muscle strength.

## 2. Methods

### 2.1. Study Design and Participants

This cross-sectional study was funded on lifestyle, markers of physical function, and anthropometric data that were collected from first-year University of Castilla La Mancha students, Spain. The sample size was estimated using the software Epidat predicting a prevalence of obesity of 22%, an alpha error of 0.05, a precision of 5%, and statistical power of 80%. Predicting a rate of not response of 20%, the sample size was 300 students. Taking as a sample frame the enrolment list, a random 560 students (aged between 18 and 30 years old) were requested, from which 360 students (64.28%) accepted to participate. To calculate the size of the sample, we have considered the obesity prevalence as an outcome variable since this study is part of the research “Lifestyle, adiposity and vascular function in college students from Castilla-La Mancha, Spain”. Finally, 355 participants were included in our analysis, as they had data on all determinations of interest (Figure S1 in Supplementary Material).

All participants included had to meet the following conditions: (i) not having any learning disability and (ii) not having any type of mental or physical dysfunction or any chronic disorder impeding their involvement in the measurements. Once the participants were informed verbally and in writing, they were asked to sign a consent form as a condition to participating in the study. The study protocol was approved by the Clinical Research Ethics Committee of The Virgen de la Luz Hospital in Cuenca, Spain (REG: 2016jPI1116).

### 2.2. Variables

The study variables were performed during the 2017–2018 academic course by seven trained researchers, following standardized conditions.

#### 2.2.1. Body Composition Variables

Weight was determined twice with the subject shoeless and wearing light clothes using a Seca Model 770 scale. Height was determined using a Seca Model 222 stadiometer. The subject was measured twice, shoeless and standing, with the sagittal midline at the midline of the stadiometer. Body mass index (BMI) was assessed as the weight divided by the square of the height (kg/m^2^). BMI was classified into four categories: underweight (BMI ≤ 18.4), normal weight (18.5 ≤ BMI ≤ 24.9), overweight (25 ≤ BMI ≤ 29.9), and obese (BMI ≥ 30) [21]. Fat mass percentage was assessed under supervised humidity requirements and temperature using an 8-electrode Tanita Segmental-418 bioimpedance analysis system (Tanita Corp., Tokyo, Japan). Additionally, measurements were performed after urination, before breakfast, and after a 15-min resting period. These determinations were assessed by trained nurses to decrease interobserver variability.

#### 2.2.2. Muscle Strength

The standing long jump test was employed for measuring the strength of the lower part of the body. Participants stood behind a line with their feet roughly shoulder-width apart and jumped with both feet as far as possible. The test evaluates the distance in centimeters from the starting line to the back of the student’s heels. The best of three tests was registered. A digital dynamometer with adaptable grip TKK 5401 GripD (Takeya, Tokyo, Japan) was used to determine upper body strength (handgrip strength in kilograms). The exam was completed twice with the left hand and twice with the right hand, and the best score for each hand was registered in kilograms; the mean average of the two measurements was estimated. Finally, with the information from these two exams, a muscle strength index was calculated as the sum of the standardized z-score of the standing long jump and z-score of handgrip/weight.

#### 2.2.3. Dietary Calcium Intake

A Food-Frequency Questionnaire (FFQ) [22] was used to calculate the total consumption of calcium. This validated questionnaire contains 137 items with 9 consumption frequencies (never or almost never, 1–3 times/month, once/week, 2–4 times/week, 5–6 times/week, once/day, 2–3 times/day, 4–6 times/day, and more than 6 times/day). Dietary calcium consumption and total energy intake were computed using Spanish food composition tables [23].

#### 2.2.4. Family Socioeconomic Status

The family socioeconomic status was assessed using the Spanish Epidemiology Society Scale [24], determining an index of socioeconomic status based on the level of occupation and education of parents. The parents’ occupation was organized independently as: (1) supervisor/manager or self-employed with 10 or more employees; (2) supervisor/manager or self-employed with fewer than 10 employees; (3) self-employed without employees; (4) nonqualified worker; and (5) housewife, unemployed, and others. The parents’ education was classified into 3 categories: (1) primary education (functional illiterates, no education, or those who did not complete primary education); (2) secondary education (primary education, secondary education, or Spanish Bachillerato’s degree); and (3) higher education (undergraduate, masters, or doctoral degrees).

### 2.3. Statistical Analyses

Descriptive characteristics and differences by sex were calculated with Student’s t-test (continuous variables: age, weight, height, % fat mass, BMI, handgrip strength, standing long jump, muscle strength index, calcium, total EI, carbohydrate %, protein %, fat %, socioeconomic status) or chi-squared test (categorical variables: BMI and fat mass categories). Graphical methods (normal probability plots) and Kolmogorov-Smirnov tests were employed to assess the normal distribution of continuous variables. All continuous variables analyzed fit a normal distribution.

The Pearson correlation coefficient was calculated to determine the relationship between dietary calcium intake, fat mass percentage, BMI, muscle strength components (handgrip strength, standing long jump, muscle strength index), and total energy intake.

ANCOVA models were used to analyze the mean differences in the muscle strength index as a dependent variable by total dietary calcium intake categories as follows: low (first quartile), medium (second and third quartile), and high (fourth quartile), controlling for age and sex (model 1). Additionally, ANCOVA models were also used to examine the mean differences in fat mass percentage as a dependent variable by total dietary index and muscle strength index categories: low (first quartile), medium (second and third quartile) and high (fourth quartile), controlling for age, sex, total energy intake and muscle strength index, and controlling for age, sex, total energy intake, and total dietary calcium intake.

Finally, a simple mediation analysis was estimated to analyze if the association between total dietary calcium intake and the fat mass percentage was mediated by the muscle strength index. In these analyses, path a symbolizes the regression coefficient between the muscle strength index (mediator) and the dietary calcium intake;(path c, total effect, was the regression coefficient of fat mass percentage on the dietary calcium intake. Path b symbolizes the regression coefficient between the muscle strength index and the fat mass percentage. The relation of the dietary calcium intake with the fat mass percentage and the muscle strength index concurrently was assessed in the path c’, direct effect. To test the statistical significance of the mediation effect, we determined the Sobel test. This test was analyzed as the ratio of the indirect effect (path a × path b) by its standard error, and a Z test was performed under the null hypothesis that the indirect effect was equal to zero [25]. Additionally, the percentage of mediation was analyzed as follows: (indirect effect/total effect) × 100. As recommended by Preacher and Hayes [26,27], the following criteria were established to determine the mediation: the independent variable must be significantly related to the mediator and dependent variables; the mediator must be significantly related to the dependent variable; the association between the independent and dependent variables must be attenuated when the mediator is introduced into the regression model.

The results showed unstandardized beta coefficients, 95% confidence intervals, and the standard error of beta estimates. For these analyses, we used PROCESS SPSS Macro version 3.1, selecting Model 4, and 5000 bias-corrected bootstrap samples. This analysis was controlled for age and sex.

Analyses were conducted using the statistical software package IBM SPSS Statistics 24.0 (SPSS Inc., Chicago, IL, USA), and the statistical significance was set at two-tailed *p* < 0.05.

## 3. Results

Table 1 shows the descriptive characteristics of the study sample. Of the 355 young adults included in the study, 125 were men (41.40%). Participants who agreed to participate did not differ in age, sex, or parenteral socioeconomic status from those who did not.

Table 2 shows the Pearson bivariate correlation coefficients between dietary calcium intake, fat mass percentage, BMI, muscle strength components (handgrip strength, standing long jump, and muscle strength index), and total energy intake. Dietary calcium intake was negatively correlated with fat mass percentage (r = −0.153; *p* < 0.001) and positively correlated with standing long jump (r = 0.149; *p* < 0.05), muscle strength index (r = 0.188; *p* < 0.001), and total energy intake. Otherwise, muscle strength components were also negatively associated with fat mass percentage and positively associated with dietary calcium and total energy intake (r = 0.883; *p* < 0.001).

**Figure 1:**
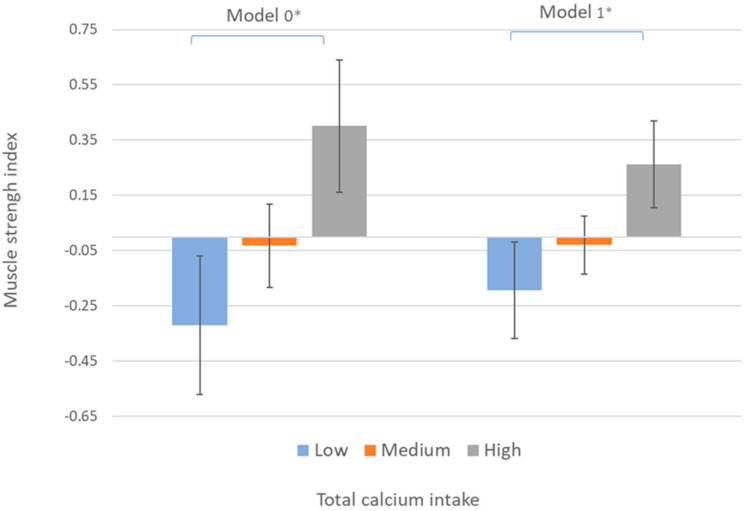
Mean differences in muscle strength index by total dietary calcium intake categories. Categories of total dietary calcium intake are low (first quartile), medium (second and third quartiles), and high (fourth quartile). Error bars represent standard error. Brackets indicate significant differences in mean (*p* < 0.05) between categories according to the Bonferroni multiple comparison post hoc test. * *p* < 0.05. Model 0: crude data. Model 1: adjusted by age and sex.

Figure 1 shows the crude mean differences in the muscle strength index by dietary calcium intake categories (model 0) and after controlling for sex and age (Model 1). Those with high calcium intake showed significantly higher muscle strength index values than their counterparts belonging to the lowest category of calcium intake.

**Figure 2:**
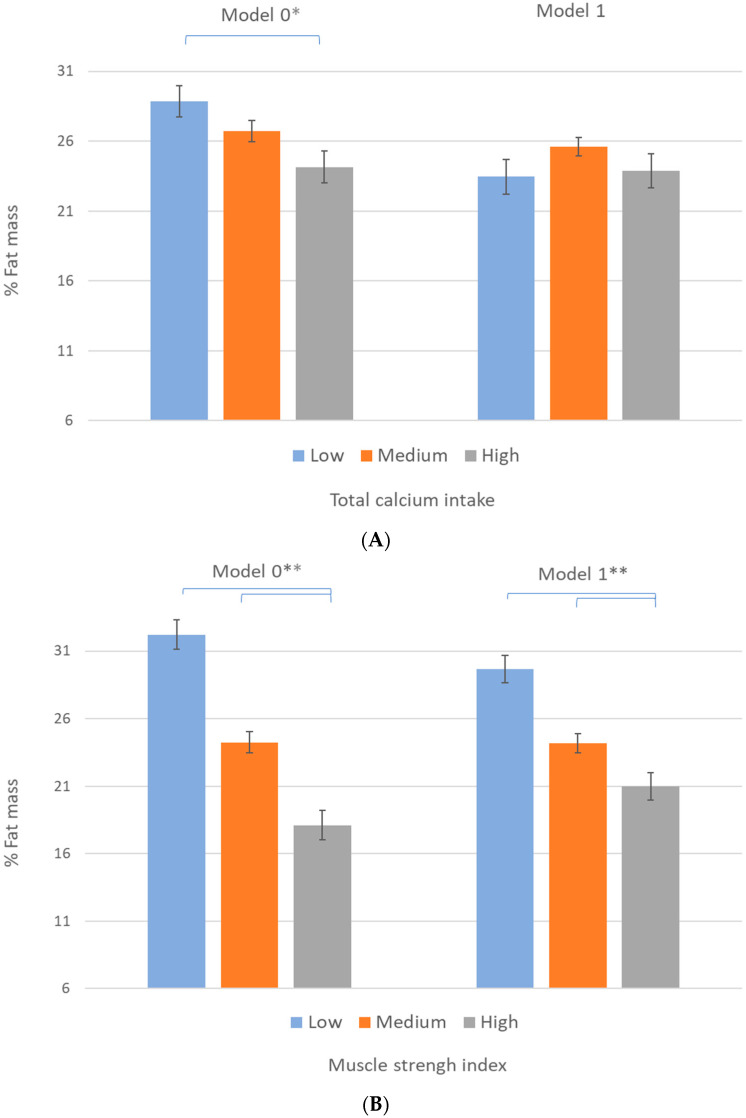
Mean differences in % fat mass by (**A**), total dietary calcium intake, and (**B**) muscle strength index categories. Categories of total dietary calcium intake and muscle strength index are low (first quartile), medium (second and third quartiles), and high (fourth quartile). Error bars represent standard error. Brackets indicate significant differences in mean (*p* < 0.05) between categories according to the Bonferroni multiple comparison post hoc test. ** *p* < 0.001, * *p* < 0.05. Model 0: crude data. Model 1 (**A**): adjusted by age, sex, total energy intake, and muscle strength index. Model 1 (**B**): adjusted by age, sex, total energy intake, and total dietary calcium intake.

Figure 2A depicts the crude mean differences in the fat mass percentage by dietary calcium intake categories. Those categorized as high calcium intake showed significantly lower values of fat mass percentage. However, when we adjusted for potential confounders, including the muscle strength index, these differences disappeared (Model 1), suggesting a potential mediating effect of the muscle strength index in its relationship with calcium intake and fat mass percentage. Additionally, Figure 2B shows the crude and adjusted mean differences in the fat mass percentage by muscle strength index categories. In both models, participants categorized as high muscle strength index showed significantly lower values in fat mass percentage than those categorized as low and medium muscle strength index.

**Figure 3:**
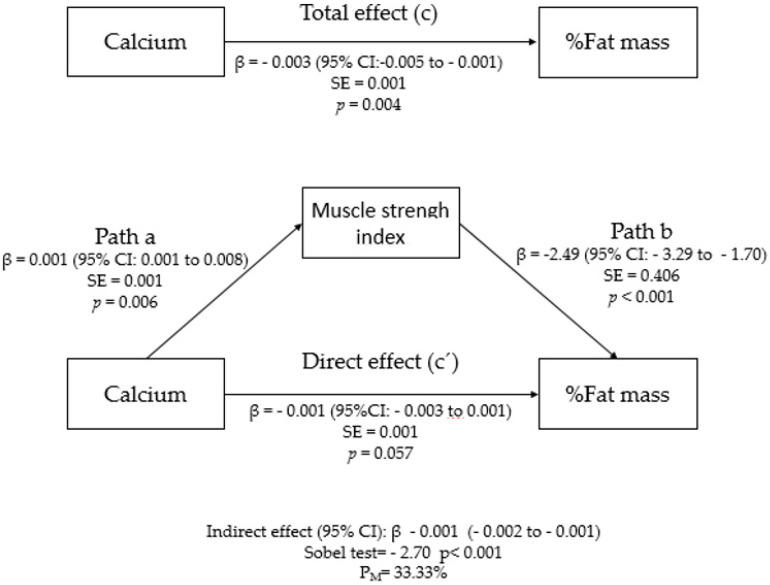
Mediation model of the relationship between total dietary calcium intake and % fat mass using muscle strength index as a mediator and controlling for sex and age. The data are presented as unstandardized beta coefficients, 95%CI, and standard error of beta estimates. Abbreviations: % fat mass, fat mass percentage; P_M_, percentage of mediation.

The model examining whether the muscle strength index mediates the association between dietary calcium intake and fat mass percentage is shown in Figure 3. In the first regression equation, calcium intake was positively correlated with the muscle strength index (path a = 0.001; 95% CI, 0.001–0.008; *p* = 0.006). In the second equation, calcium intake was negatively correlated with fat mass percentage (path c = −0.003; 95% CI, −0.005 to −0.001; *p* = 0.004). However, in the third equation, the association between calcium intake and the fat mass percentage was reduced when the mediator (muscle strength index) was incorporated into the model (path c’= −0.001; 95% CI, −0.003 to 0.001; *p* = 0.057). In this sense, these results suggest that the relationship between calcium intake and the fat mass percentage was completely mediated by the muscle strength index, as confirmed by the values shown with the Sobel test (z = −1.90, *p* < 0.001). The percentage of mediation was 33.33%.

## 4. Discussion

This study produced three main findings when examining the role of dietary calcium intake and muscle strength in managing excess fat mass in young adults. First, dietary calcium intake and muscle strength index showed inverse associations with fat mass percentage. Second, dietary calcium intake has a positive influence on muscle strength. Third, the muscle strength index acts as a total mediator in the relationship between dietary calcium intake and fat mass percentage.

It is well known the important role of calcium in muscle contraction. According to previous evidence [28], our data indicate higher results of muscle strength in young adults with a high level of dietary calcium intake; hence, it is plausible that an adequate calcium intake is considered. Notwithstanding, the influence of vitamin D or calcium supplementation on muscle strength is controversial. A previous meta-analysis has found a positive impact of vitamin D supplementation on muscle strength in people aged on average 61 years [29], but in young adults, the evidence of vitamin D/calcium supplementation has not shown a significant influence [30], even in young males where vitamin D might affect the synergy between testosterone and muscle strength [13].

Moreover, dietary calcium intake has been associated with lower adiposity in children and adolescents [19,20], and calcium supplements have been implicated in the prevention or treatment of overweight [31]. The anti-obesity mechanisms of calcium may be related to a high intracellular calcium concentration that attenuates adipocyte lipid accretion and stimulates lipolysis, promoting greater rates of fat oxidation [32]. In addition, it has been suggested that dietary calcium consumption could influence fat metabolism by increasing fecal fatty acid excretion [33]. Our data from college students are partially in line with this previous evidence because they indicate the positive effect of dietary calcium intake on reducing fat mass percentage. However, these differences regularly disappeared once we controlled for muscle strength index (Model 1), suggesting that the effect of dietary calcium intake on fat mass might be explained by the effect of muscle strength. In this sense, it has been recognized a favorable influence of exercise on weight management and body composition in adults with an excess of adiposity [34]. Modalities as circuit training interventions [35], high-intensity interval training [36], or aerobic training [37], does favor weight loss. Aerobic training also seems to be better than resistance or combined training (aerobic combined with resistant training) to this proposal [37].

The relationship between excess fat mass and muscle strength is complex. It has been shown that there is a better effect of an excess of fat mass on lower limbs muscle strength (loading musculature) than on upper limbs [38]. Moreover, individuals with an excess of adiposity have greater absolute strength, but when the measurement of strength is controlled by body weight (a relative indicator) appears weaker. Thus, adiposity could lead to non-optimal muscle contraction and reduced calcium signaling, which can adversely affect muscle strength [39]. Moreover, higher levels of muscle strength are related to lower inflammatory status, which seems to be explained by lower levels of fatness [40]. In our study, as a measure of strength, we use a relative muscle strength index, combining upper and lower limb assessments adjusted by body weight [41]. In addition, our results, agreeing with these previous findings, demonstrate that young adults with a good muscle strength index level had a lower fat mass percentage than their peers with a poor muscle strength level, even after controlling for dietary calcium intake. Therefore, it seems believable that the positive effect of dietary calcium intake on the fat mass percentage could be due to the influence of calcium on muscle strength. In this sense, our data revealed a total mediating role of the muscle strength index in the relationship between dietary calcium intake and fat mass percentage.

### Limitations

This study presents several limitations. First, the study had a cross-sectional design, which prevented us from drawing cause-effect inferences. Second, we tested the effect of dietary calcium intake and muscle strength index on fat mass percentage, but we did not consider other body fat distributions, such as subcutaneous or visceral or fat mass. Third, self-reported data of dietary calcium intake are susceptible to being affected by memory bias; thus, it could have been affected by under- or overreporting. To assess this variable, we also selected the FFQ-137 items, this questionnaire has been used in previous studies with young adults [42,43], and although it has not been validated for this population, it has been validated for other population groups from children to the elderly [22,44,45]. Fourth, the results of our study must be interpreted with caution since bioelectrical impedance analysis and DXA might be interchangeable to determine % fat mass, except in obese people [46,47] although, in our sample, the proportion of obese people is negligible. Finally, the relationship between dietary calcium intake and muscle strength could be potentially confounded by other nutritional and sociodemographic variables that were not measured and that, as a consequence, were not included in the analyses.

## 5. Conclusions

Our findings show that both dietary calcium intake and muscle strength are associated with fat mass percentage. Moreover, they support that muscle strength plays a key role in the relationship between dietary calcium intake and fat mass percentage. Therefore, promoting interventions designed to motivate young people towards leading healthy lifestyles, including high dietary calcium intake and mainly exercise activities aimed at improving muscle strength levels, may help to control an excess of fat mass and prevent cardiometabolic risk related to it.

## Figures and Tables

**Table 1:** Descriptive characteristics of the study sample by sex.

Variable	All (*n* = 355)	Men (*n* = 125)	Women (*n* = 230)	*p* *
Age (years)	21.05 ± 3.11	21.19 ± 2.85	20.97 ± 3.25	0.534
Weight (Kg)	65.41 ± 12.34	72.60 ± 10.94	61.50 ± 11.27	**<0.001**
Height (cm)	167.30 ± 8.66	175.39 ± 7.03	162.93 ± 5.86	**<0.001**
% Fat mass	26.61 ± 10.01	18.85 ± 6.83	30.68 ± 8.95	**<0.001**
Low (%) ^&^	24.9	45.7	10.1	**<0.001**
Moderate (%) ^&^	50.2	37.8	53.4	
High (%) ^&^	24.9	2.4	36.4	
BMI (Kg/m^2^)	23.28 ± 3.62	23.55 ± 3.03	23.14 ± 3.90	0.308
Underweight (%)	3.1	0.8	4.4	
Normal weight (%)	70.6	70.6	70.6	0.068
Overweight (%)	21.8	26.2	19.3	
Obesity (%)	4.5	2.4	5.7	
Handgrip strength (Kg)	30.41 ± 9.51	39.22 ± 7.75	24.43 ± 4.77	**<0.001**
Standing long jump (cm)	161.21 ± 43.78	195.49 ± 31.91	136.82 ± 33.55	**<0.001**
Muscle strength index (z-score) ^a^	0.013 ± 1.7	1.52 ± 1.25	−1.05 ± 1.21	**<0.001**
Calcium (mg/dL)	1219.77 ± 555.30	1241.32 ± 562.64	1208.07 ± 542.65	0.693
Total EI (Kcal)	2795.79 ± 1804.77	2865.92 ± 1287.02	2757.68 ± 2033.24	0.590
Carbohydrate (% EI)	43.01 ± 7.10	43.10 ± 6.63	42.95 ± 7.36	0.852
Protein (% EI)	17.47 ± 3.46	17.39 ± 3.23	17.51 ± 3.59	0.749
Fat (% EI)	38.18 ± 6.21	37.93 ± 5.98	38.32 ± 6.34	0.578
Socioeconomic status				
Low (%)	28.3	30.8	27.0	**0.048**
Medium (%)	46.6	52.1	43.7	
High (%)	25.1	17.1	29.3	

Results are presented as mean and (±) standard desviation. Bold values indicate statistical significance *p* < 0.05. Abbreviations: BMI body mass index; EI, energy intake; % Fat mass, fat mass percentage. ^&^ Low representing (1st quartile), medium (second and third quartiles), and high (4th quartile). ^a^ Sum of the standardized z score of standing long jump and dynamometry/weight. * Chi-squared tests (categorical variables) or T student tests (continuous variables).

**Table 2:** Bivariate correlations between nutrition, muscle strength, and body composition variables.

	Calcium	%Fat Mass	BMI	Handgrip Strength	Standing Long Jump	Muscle Strength Index ^a^	Total EI
Calcium	-	−0.153 **	−0.091	0.070	0.149 *	0.188 **	0.883 **
%fat mass		-	0.493 **	−0.390 **	−0.531 **	−0.632 **	−0.166 **
BMI			-	0.224 **	−0.66	−0.065	−0.116*
Handgrip strength				-	0.611 **	0.184 **	0.101
Standing long jump					-	0.783 **	0.206 **
Muscle strength index ^a^						-	0.243 **

Data are presented in the correlation coefficient R. * *p* < 0.05, ** *p* < 0.001. Abbreviations: BMI, body mass index; EI, energy intake; %fat mass, fat mass percentage Data are shown as the correlation coefficient R. * *p* < 0.05, ** *p* < 0.001. ^a^ Sum of the standardized z-score of standing long jump and dynamometry/weight.

## Data Availability

The datasets generated during and/or analyzed during the current study are available from the corresponding author (Vicente Martínez Vizcaíno) on reasonable request.

## Supplementary Materials

The following are available online at https://www.mdpi.com/article/10.3390/nu13124498/s1, Figure S1: Diagram describing participants enrolment.
