# Supplementary figures and images for: Dietary Calcium Intake and Fat Mass in Spanish Young Adults: The Role of Muscle Strength
Source: Nutrients. 2021 Dec 16;13(12):4498. doi: 10.3390/nu13124498 (PMC8705271; doi:10.3390/nu13124498)

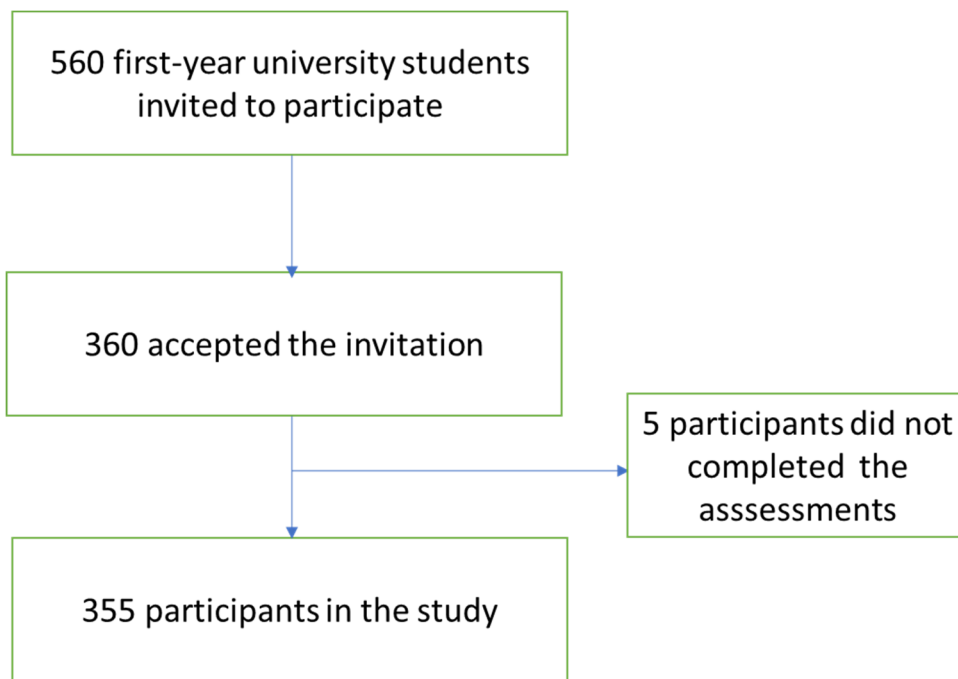

Figure S1. Diagram describing participants enrolment

Supplement: Supplementary file 1 [file nutrients-13-04498-s001.zip › nutrients-1491075-supplementary.pdf]
